# Aloe polysaccharide promotes osteogenesis potential of adipose-derived stromal cells via BMP-2/Smads and prevents ovariectomized-induced osteoporosis

**DOI:** 10.1007/s11033-022-08003-x

**Published:** 2022-10-15

**Authors:** Xue-wei Yao, He-dong Liu, Mao-xian Ren, Tian-lin Li, Wen-kai Jiang, Zhi Zhou, Zhi-yi Liu, Min Yang

**Affiliations:** Department of Trauma Orthopedics, The First Affiliated Hospital of Wannan Medical College, Yijishan Hospital, No.2, Zheshan Xi Road, Wuhu, 241001 Anhui People’s Republic of China

**Keywords:** BMP-2/Smads signaling pathway, Adipose-derived stromal cells, Aloe polysaccharide, Osteoporosis

## Abstract

**Background:**

Aloe polysaccharide (AP) is a type of an active macromolecule of Aloe vera, which contributes to its function. However, whether AP possesses anti-osteoporosis properties is unknown.

**Methods:**

Adipose-derived stromal cells were treated with different concentrations of AP. Early and late osteogenesis were, respectively, evaluated by ALP and Alizarin Red S staining. The effect of AP on the processes of adipogenesis inhibition in ADSCs was analyzed by oil red O staining. Western blot was used to assess the expression of osteogenic and adipogenic related factors. Then, Noggin was administered to further confirm the mechanism by which AP promotes the osteogenesis of ADSCs. Finally, 40 female SD rats were classified into a bilateral laparotomy group (Sham group) and three bilateral ovariectomy groups: OVX group, OVX + AP group, and OVX + AP + Noggin group. The bilateral rat femurs were collected to perform micro-CT scanning, HE, Masson trichrome, and Oil red O staining.

**Results:**

The results indicated that AP could increase ALP expression and calcium deposition. Through molecular mechanisms, AP promotes the protein expression of COL1A1, OPN, and ALP in ADSCs, but downregulates the expression of PPARγ. Also, AP directs ADSCs’ fate by stimulating the BMP2/Smads signaling pathway. In vivo, the rat AP-treated had more trabecular bone than the OVX rat, indicating partial protection from cancellous bone loss after treatment with AP.

**Conclusion:**

Our results show that AP may promote osteogenesis of ADSCs through BMP-2/Smads signaling pathway and inhibits lipogenic differentiation. Thus, AP might be a promising alternative medicine to treat postmenopausal osteoporosis.

## Introduction

Osteoporosis is a prevalent illness marked by a loss of bone mineral density and degeneration of bone microarchitecture, as well as an increased risk of fracture [1]. According to an EU epidemiological survey, 27.7 million people have osteoporosis, and economic losses from fractures are estimated to be 37 billion euros, with expenses expected to rise by 25% by 2025 [2]. At present, the treatment of osteoporosis includes bisphosphonates, raloxifene, selective estrogen receptor modulators (SERMs), estrogens, and calcitonin [3]. However, the application of these drugs in clinical use is limited by their adverse side effects. Adverse effects that have been mentioned include subtrochanteric femoral fractures [4], osteonecrosis of the long bones and jaws [5], breast carcinoma, endometrial cancer, thromboembolic events, and cardiovascular disease [6]. Therefore, natural sources with fewer side effects but significant curative effects have attracted extensive attention.

Nondigestible bioactive polysaccharides are prominent for preventing osteoporosis and bone diseases with great efficiency. Polygonatum sibiricum polysaccharide when passed through the ERK/GSK-3 β/β-catenin pathway promotes osteoblast differentiation and prevents bone loss [7]. Aloe vera belongs to the Liliaceae family and is rich in diverse bioactive substances [7]. Products of Aloe vera, whether as juice, fresh gel, or formulated products, have been also used for medical, health, and cosmetic purposes over the years [8]. As the principal bioactive component in aloe vera [9], aloe polysaccharide (AP) is a kind of active macromolecular compounds [10]. And AP has been proved to have the same properties as other polysaccharides, such as antioxidant [11], anti-inflammatory [12], immunomodulation [13], antimicrobial [14], antiviral, and anticancer [15]. However, the direct application of AP in osteoporosis model and its effect on osteogenic and adipogenic differentiation of ADSCs have not been reported.

Under specific conditions and environments, Adipose-derived stromal cells (ADSCs) has multidirectional differentiation potential and can differentiate into adipocytes, osteoblasts, and chondrocytes [16]. The adipogenic and osteogenic lineages of ADSC differentiation are mutually inhibitory, with adipogenic commitment blocking osteogenic differentiation and vice versa [17]. Under the condition that a large number of cells are required for regeneration, the abundance, easy access, and low cytotoxicity make ADSCs a better substitute for BMSCs [18]. Specifically inhibiting ADSC adipogenesis while simultaneously promoting ADSC osteogenesis could be a potential treatment for osteoporosis [19].

Therefore, it’s worth further defining the effects of AP on osteoporosis. In this study, we tested the hypothesis that AP prevents estrogen deficiency-induced bone loss and promotes osteogenic and inhibits lipogenic differentiation of ADSCs, and tried to provide new evidence for AP can be used as a promising drug for the prevention and treatment of osteoporosis.

## Materials and methods

### ADSC isolation and culture

The bilateral inguinal fat was isolated from young SD rats in a sterile environment, cut into 1 mm³ size, and washed with 1 × PBS. Then, 0.1% type I collagenase in the same volume was mixed into the tissue and digested in a water bath at 37 °C for 1 h. During this period, the tissue vibrated every 10 min. The mixture was strained through a 100 µ M cell sieve to remove undigested tissues before being centrifuged at 1200 rpm for 15 min. The cells were resuspended in a complete medium after discarding the supernatant. Then, the cells were cultured at 37 °C in a 5% CO_2_ cell incubator, until the cell density reaches 70–80%. Third passage cells were used in all of the studies. A complete medium is a DMEM high glucose medium (HyClone, USA) that has been supplemented with 10% FBS (Gibco, USA) and 1% penicillin-streptomycin (Beyotime, China).

### Identification of ADSCs

The ADSCs were identified by flow cytometry. The cells were harvested with 0.25% trypsin and centrifuged at 1000 rpm for 5 min. Then, 1 × 10^6^ cells were resuspended in PBS and incubated using the following antibodies at 4 °C: CD29, CD90, CD31, and CD45 (Biolegend, USA).

To confirm the multipotency of ADSCs, they were subjected to induced differentiation by culturing them in osteogenic induction medium (OIM, Cyagen Biosciences, USA), adipogenic induction medium (AIM, Cyagen Biosciences, USA), and chondrogenic induction medium (Cyagen Biosciences, USA) and then verified with Alizarin Red, Oil Red O, and toluidine blue staining, respectively.

### Cell proliferation assay

ADSCs were cultured in 96-well plates at a density of 2 × 10^3^ cells/well for 24 h before being cultured with a complete medium containing AP (purity ≥ 98%; purchased from Xi’an Zelong Biotechnology Co., Ltd, contains mannose-6-phosphate, glucose, galactose, rhamnose, etc.) at concentrations of 0, 25, 50, 100, 200, and 400 µg/L. Cell proliferation was detected by a Cell Counting Kit-8 (CCK-8, Solarbio, China) on days 1, 3, and 7. In a nutshell, 96-well plates were washed with 1 × PBS, and then, 100 µL of a high glucose medium and 10 µL of the color developer were added into each well. After incubation at 37 °C in a 5% CO_2_ incubator for 2 h, the absorbance in each group was recorded at a wavelength of 450 nm by a microplate reader (Bio Tek Instruments, Inc; USA), and the cell proliferation rate was detected according to the product manual steps.

### Alkaline phosphatase (ALP) and alizarin red S staining (ARS)

ADSCs were seeded in 6-well plates (1 × 10^5^ per well). When the cell density reaches 70–80%, the osteogenic induction medium is changed every 3 days. For ALP staining, ADSCs were fixed with 4% paraformaldehyde on day 7 and rinsed with 1 × PBS. The osteogenic differentiation ability was tested by the BCIP/NBT chromogenic substrate (Beyotime, China). The images were captured under a microscope after washing with 1 × PBS. To assess ALP activity, the cells were lysed by RIPA cell lysate (Beyotime, China), and the supernatant was taken after centrifugation. Analysis of ALP activity was performed using an Alkaline Phosphatase Assay Kit (Beyotime, China). The absorbance in each group was recorded at a wavelength of 405 nm by a microplate reader (Bio Tek Instruments, Inc; USA).

To test the bone mineralization ability of ADSCs, they were treated in the same way for 21 days. ADSCs were fixed with 4% paraformaldehyde and then rinsed with 1 × PBS. The ADSCs were stained with 0.2% ARS staining (Solarbio, China) at room temperature. The stained cells were captured by a microscope after 30 min of washing with 1 × PBS. To quantify mineralization, the Alizarin red dye was eluted with 10% cetylpyridinium chloride (Solarbio, China), and the absorbance in each group was recorded at a wavelength of 570 nm by a microplate reader (Bio Tek Instruments, Inc; USA).

### Oil red O staining

ADSCs were seeded in six-well plates (1 × 10^5^ per well). When the cell density reached 100%, the adipogenic induction medium was changed every 3 days. ADSCs were fixed with 4% paraformaldehyde on day 21 and rinsed with 1×PBS. The cells were stained using oil red O (Solarbio, China) and viewed under a light microscope.

### Western blotting

After 14 days of osteogenesis or adipogenesis, the ADSCs were lysed with the RIPA cell lysate and protease inhibitor to extract the total protein. SDS-PAGE (Solarbio, China) was used to separate proteins with various molecular weights, and then, the separated proteins were blotted onto a PVDF membrane (Millipore, USA) using a transBlot turbo transfer system. After being sealed in 5% skimmed milk for 2 h, the membrane was incubated in the primary antibody overnight at 4 °C. The primary antibodies used are as follows: anti-COL1A1 AF7001, Affinity, USA, 1:1000), anti-ALP (DF6225, Affinity, USA, 1:1000), anti-OPN (AF0227, Affinity, USA, 1:2000), anti-BMP2 (AF5163, Affinity, USA, 1:1000), anti-RUNX2 (AF5185, Affinity, USA, 1:2000), anti-β-actin (AF7018, Affinity, USA, 1:10,000), anti-p-SMAD1/5/8 (AF8313, Affinity, USA, 1:2000), and anti-PPARγ (AF6284, Affinity, USA, 1:2000). Then, the membrane was incubated with the HRP-conjugated second antibody for 2 h and detected by ECL and super signal detection agents (Thermo Fisher Scientific, USA).

### Animals studies

Forty female Sprague-Dawley rats, weighing 260 ± 10 g (2 months old), were used for the experiments. All rats were kept (4 per cage) under standard ventilation, 12 h light–dark cycle, temperature (25 ± 3 °C), and humidity (55 ± 5%). The rats were fed with adequate water and food. As described previously [20, 21], all rats were fixed in the prone position and given either a bilateral laparotomy (Sham, n = 10) or a bilateral ovariectomy (OVX, n = 30) after 7 days of acclimatization. Then, all rats were anesthetized with pentobarbital sodium (100 mg/kg) before the operation, and all the operations followed aseptic techniques. Penicillin (10 × 104 U) was injected intramuscularly postoperatively each day for 3 days.

After 4 weeks, the rats were randomly classified into four groups: Sham (n = 10) and OVX (n = 10) received normal saline at a concentration of 50 ml/kg.d; OVX + AP group (n = 10) received aloe polysaccharide (300 mg/kg.d); and OVX + AP + Noggin group (n = 10) received aloe polysaccharide (300 mg/kg.d) with Noggin (100 ng). Rats were gavaged with AP and intraperitoneal injections of noggin once daily for 12 weeks. The dose of AP was chosen based on previous studies with minor modifications [22].

### Microcomputed tomographic (micro-CT) analysis

All of the rats were killed after 12 weeks. The left and right femurs were collected, cleansed of the muscles and fascia around the femur, and fixed in 10% formalin for testing. To determine the trabecular bone parameters of the left femur, histomorphometric analyses were performed on a microcomputed tomographic (micro-CT) scanner. 3D images were reconstructed using the manufacturer’s software. Histomorphometric parameters, including bone volume/total volume (BV/TV), trabecular thickness (Tb. Th), trabecular number (Tb. N), trabecular separation (Tb. Sp), and bone mineral density (BMD), were determined with built-in software.

### Histology

For decalcification of the right femur, 10% ethylenediaminetetraacetic acid (EDTA) was used and replaced once a week for 8 weeks. The right femur was then rinsed with running water for 24 h and soaked in a series of graded concentrations of alcohol. Then, they were embedded in paraffin and incised into 4 μm slices. The samples were stained with H&E and Masson’s dye. ImageJ software (version 1.52v, NIH, USA) was used to measure the new bone formation in the distal femur. At the same time, some femurs were stained with oil red O through frozen sections.

### Statistical analysis

All the data were compiled using GraphPad Prism version 8 software (USA). Numerical data were expressed as mean ± standard deviation. One-way ANOVA followed by Tukey’s post hoc test was used to compare differences within groups. P < 0.05 was considered significantly different.

## Results

### Cultivation and characterization of rat ADSCs

**Fig. 1:**
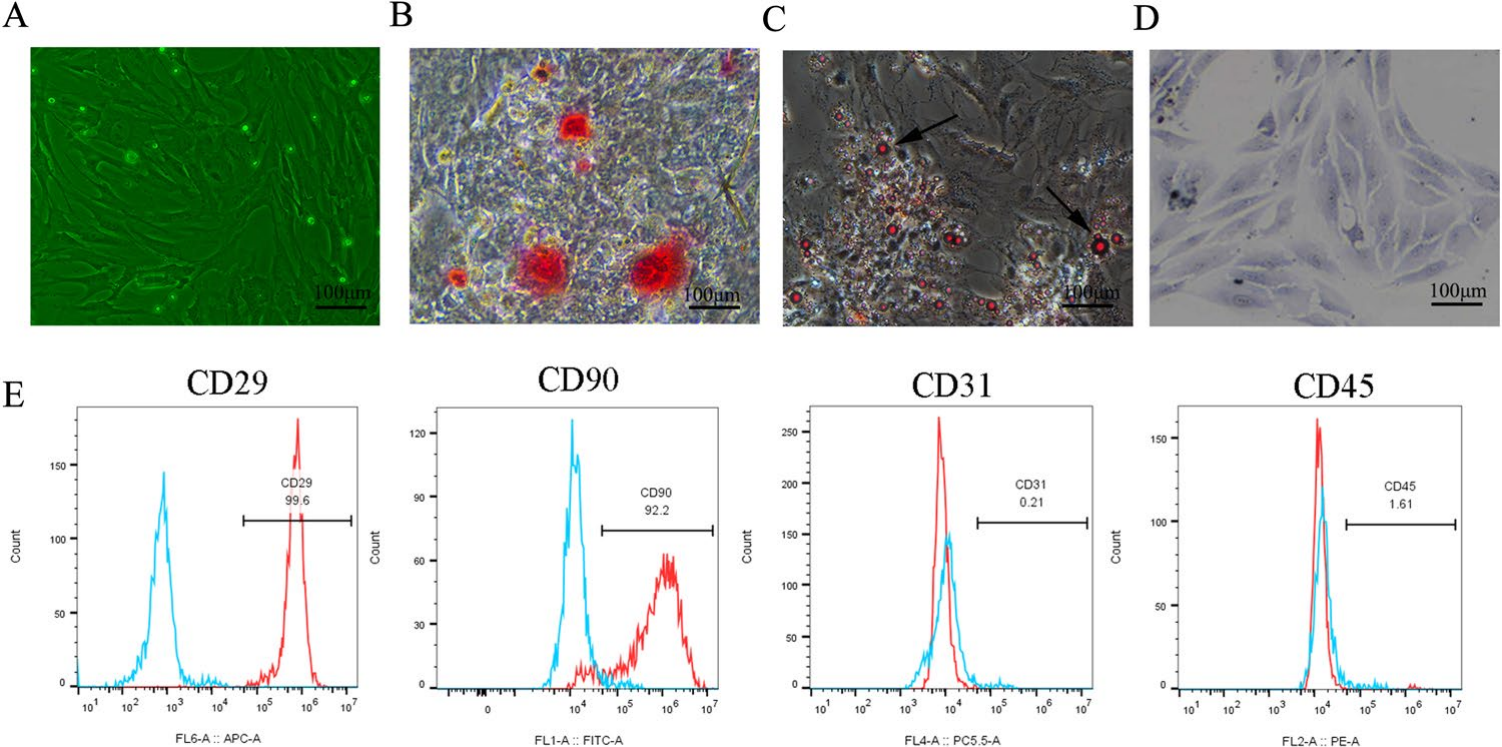
ADSC isolation, culture, and identification. **a** ADSCs had a typical spindle shape. **b–d** Alizarin Red, Oil Red O, and Toluidine Blue staining were positive after the induced differentiation of ADSCs. Scale bar = 100 μm. **e** ADSCs were positive for CD29 and CD90 while negative for CD31 and CD45. Data are presented as the mean ± SD (n = 3)

ADSCs isolated from rats had a typical spindle shape (Fig. 1a). To analyze the differentiation potential of ADSCs, they were induced into osteogenesis, adipogenesis, and chondrogenesis. Alizarin Red (Fig. 1b), Oil Red O (Fig. 1c), and Toluidine Blue staining (Fig. 1d) were positive after the induced differentiation of ADSCs. Most of ADSCs expressed CD29 and CD90. In contrast, the majority of the cells were negative for CD31 and CD45 (Fig. 1e).

### Effects of AP on proliferation and osteogenic differentiation of ADSCs

**Fig. 2:**
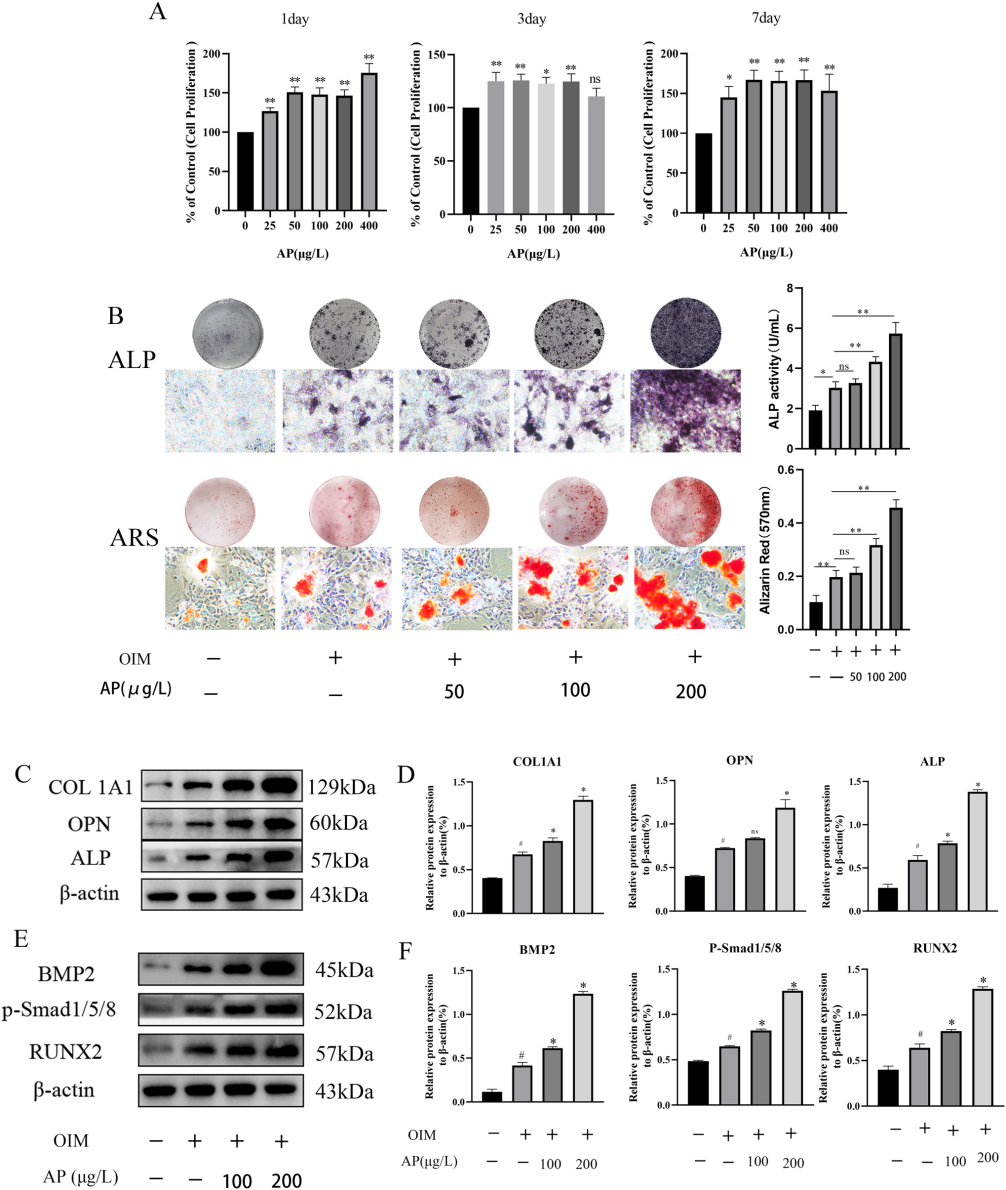
Effects of AP on proliferation and osteogenic differentiation of ADSCs. **a** The effect of AP on ADSC proliferation. **b** AP enhances ALP activity and extracellular mineralization in osteo-induced ADSCs. **c**, **d** Representative western blots and quantitative analysis of COL1A1, OPN, and ALP protein expression in different treatment groups. **e**, **f** Western blotting analyses of the protein abundance of BMP2, p-Smad1/5/8, and RUNX2 in different treatment groups. Scale bar = 100 μm. #P < 0.01 versus untreated group. Data are presented as the mean ± SD (n = 3). *P < 0.05 and **P < 0.01

To verify the effect of AP on ADSCs proliferation, ADSCs were treated at different concentrations of AP and time gradient. Figure 2a shows that AP (25, 50, 100, and 200 µg/L) has a promoting effect on cell proliferation. However, the proliferation of ADSCs decreased slightly on days 3 and 7 when the concentration of AP was 400 µg/L.

As shown in Fig. 2b, osteogenic induction group could enhance ALP activity and extracellular calcium deposition compared to untreated group. In the presence of AP, low concentration of AP did not significantly promote the osteogenic differentiation of ADSCs. When AP concentration reaches 100 and 200 µg/L, ALP activity was 4.33 and 5.73 U/ml respectively, the difference was statistically significant compared to the osteogenic induction group. At the same time, alizarin red staining has the result of acquaintance.

### AP regulates the differentiation of ADSCs into osteoblasts in vitro

To study the induction function of AP on osteogenesis of ADSCs, we induced ADSCs with AP in vitro at the concentrations of 100 or 200 µg/L. From Western blot, the expression of osteogenic-related proteins such as COL1A1, ALP, and OPN was elevated in AP-treated ADSCs, demonstrating that AP regulates the differentiation of ADSCs into osteoblasts (Fig. 2c).

### BMP-2/smads signaling is activated by the treatment of AP

The BMP-2/Smads signaling is a crucial part of bone formation and development. We identify the expression of BMP-2/Smad signaling pathway-associated proteins to learn more about how AP supports the differentiation of ADSCs into osteoblasts. In comparison to the blank group, the expression of BMP2, p-Smad1/5/8, and RUNX2 was all elevated in the other groups (P < 0.05) (Fig. 2e). Furthermore, the 200 µg/L AP-treated group showed the greatest rise. These findings indicate that AP induced the activation of BMP-2/Smad signaling pathways in ADSCs.

### Silencing BMP2 partially reversed AP regulation on the differentiation of ADSCs into osteoblasts

**Fig. 3:**
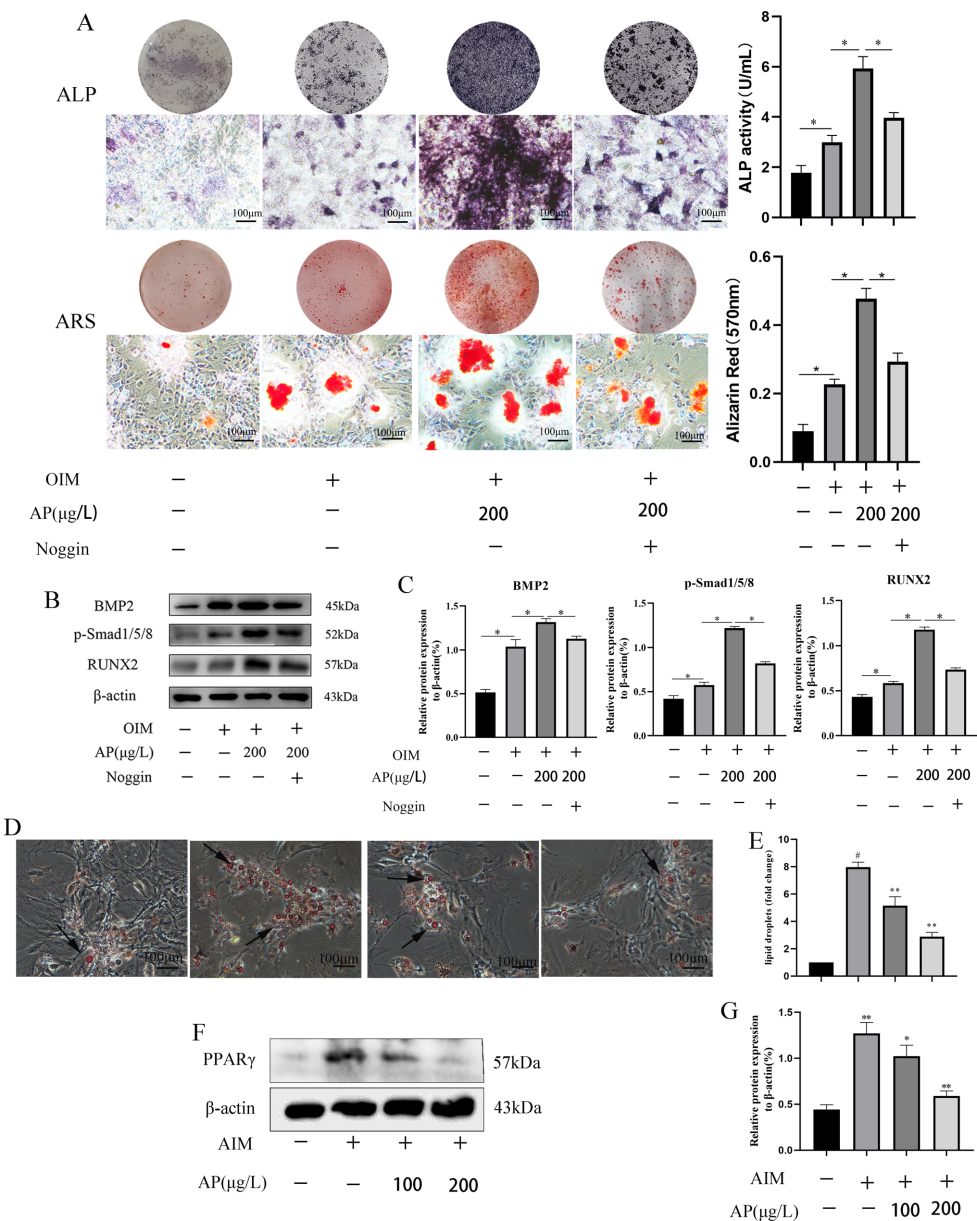
Silencing BMP2 partially reversed AP regulation on the differentiation of ADSCs into osteoblasts. **a** ALP and ARS staining in different groups. **b** Western blot analyzed the BMP-2/Smads pathway in different groups. Values in **c** represent the fold change compared to the control. **d**, **e** ADSCs were induced to differentiate by the adipogenic induction medium in the presence or absence of AP (100 and 200 µg/L), and the extent of lipid droplet aggregation within the cells was observed by oil red O staining. **f** Representative western blots. **g** Quantitative analysis of PPARγ protein expression level in different treatment groups. Data are presented as the mean ± SD (n = 3). Scale bar = 100 μm. *P < 0.05 and **P < 0.01

To further confirm that AP promotes the osteogenic differentiation of ADSCs through the BMP-2/Smads signaling pathway, we used Noggin as a BMP2 inhibitor. As shown in Fig. 3a, silencing BMP2 partially reversed the staining area and density of mineralization and calcification of ADSCs by AP. Western blot results also confirmed the above results. The expression of BMP2, p-smad1/5/8, and Runx2 was downregulated in the Noggin group than in the OIM + AP group (Fig. 3b).

### Effects of AP on the adipogenic differentiation of ADSCs

Adipogenic differentiation of ADSCs was studied under AP treatment. Differentiation of ADSCs into adipocytes was induced in the absence and presence of AP (100 and 200 µg/L). Staining of intracellular lipid droplets by Oil Red O confirmed the differentiation of ADSCs toward mature adipocytes. Figure 3d shows that AP at concentrations of 100 and 200 µg/L inhibits adipogenic differentiation of ADSCs and reduces the production of lipid droplets in cells. To investigate the effect of AP treatment on the expression of a key adipogenic marker (PPARγ), parallel effects were observed in Oil Red O dyeing and protein (Fig. 3f) expression of adipogenic markers in AP-treated ADSCs.

### Micro-CT analysis of osteoporotic rat femurs

**Fig. 4:**
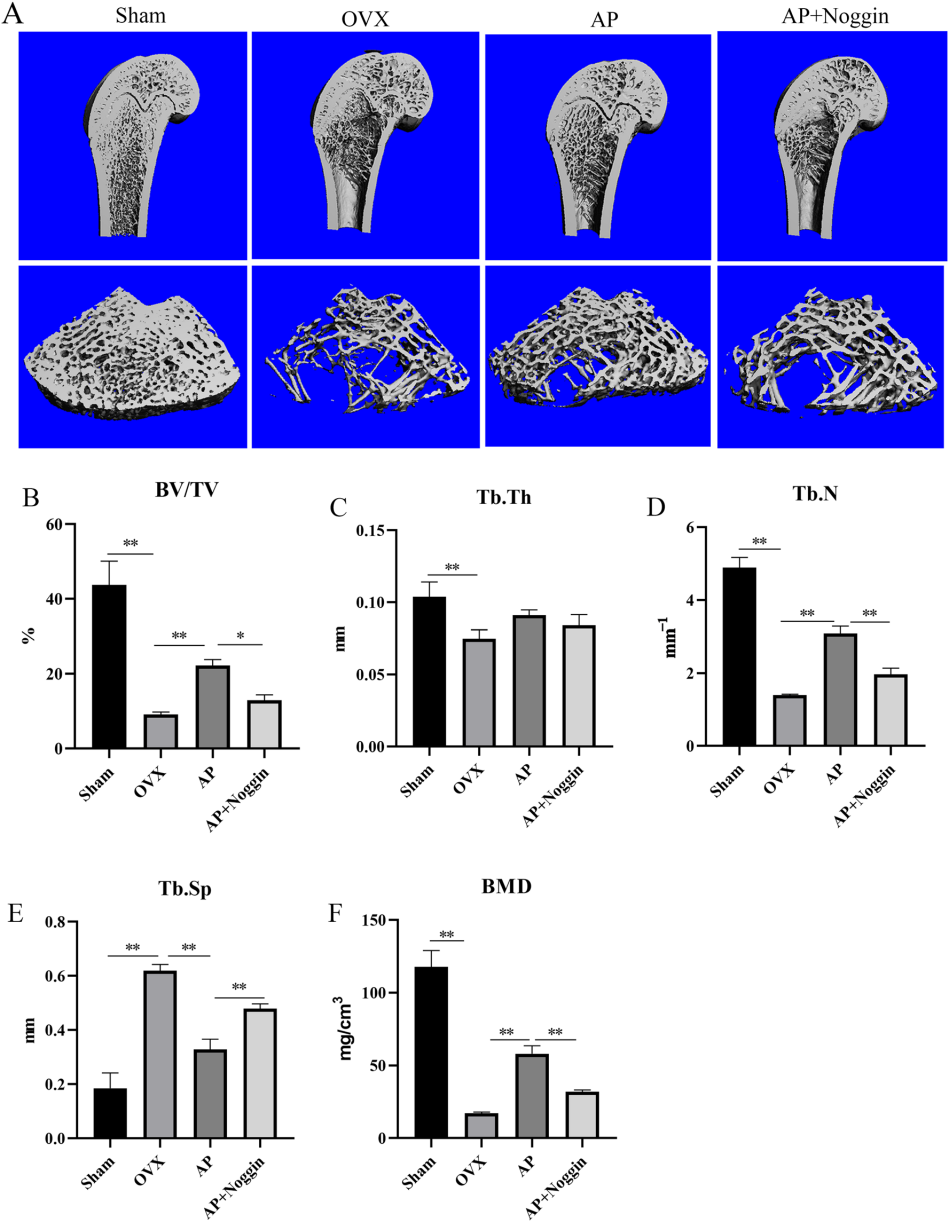
Role of AP on bone loss in estrogen-deficient rats. **a** Micro-CT scanning results of femurs in four groups of rats. **b** BV/TV, bone volume/total volume. **c** Tb. Th, trabecular thickness. **d** Tb. N, trabecular number. **e** Tb. Sp, trabecular separation. **f** BMD, Bone mineral density. Data are presented as the mean ± SD (n = 3). *P < 0.05 and **P < 0.01

Micro-CT is supposed to be a great demonstration of bone trabecular microstructure. As shown in Fig. 4a, the OVX group had fewer distal femur trabeculae bones in comparison to the Sham group. The number of bone trabeculae recovered significantly after varied doses of AP treatment, and bone density and integrity were improved. However, this effect could be inhibited by Noggin.

Micro-CT data analysis of representative samples showed that in comparison to the Sham group, the values of trabecular bone BV/TV (Fig. 4b), Tb.Th (Fig. 4c), Tb. N (Fig. 4d), and BMD (Fig. 4f) were significantly downregulated in the OVX group (p < 0.01). In comparison, the value of trabecular bones Tb.Sp was upregulated (p < 0.01) in the OVX group (Fig. 4e). In addition, the AP treatment group protected against trabecular bone deterioration by reversing the adverse effects of ovariectomy. Interestingly, when noggin was added, this effect is partially neutralized. These results indicated that AP can prevent the deterioration of bone trabecular microstructure and the occurrence of osteoporosis in OVX rats.

### Histological evaluation

**Fig. 5:**
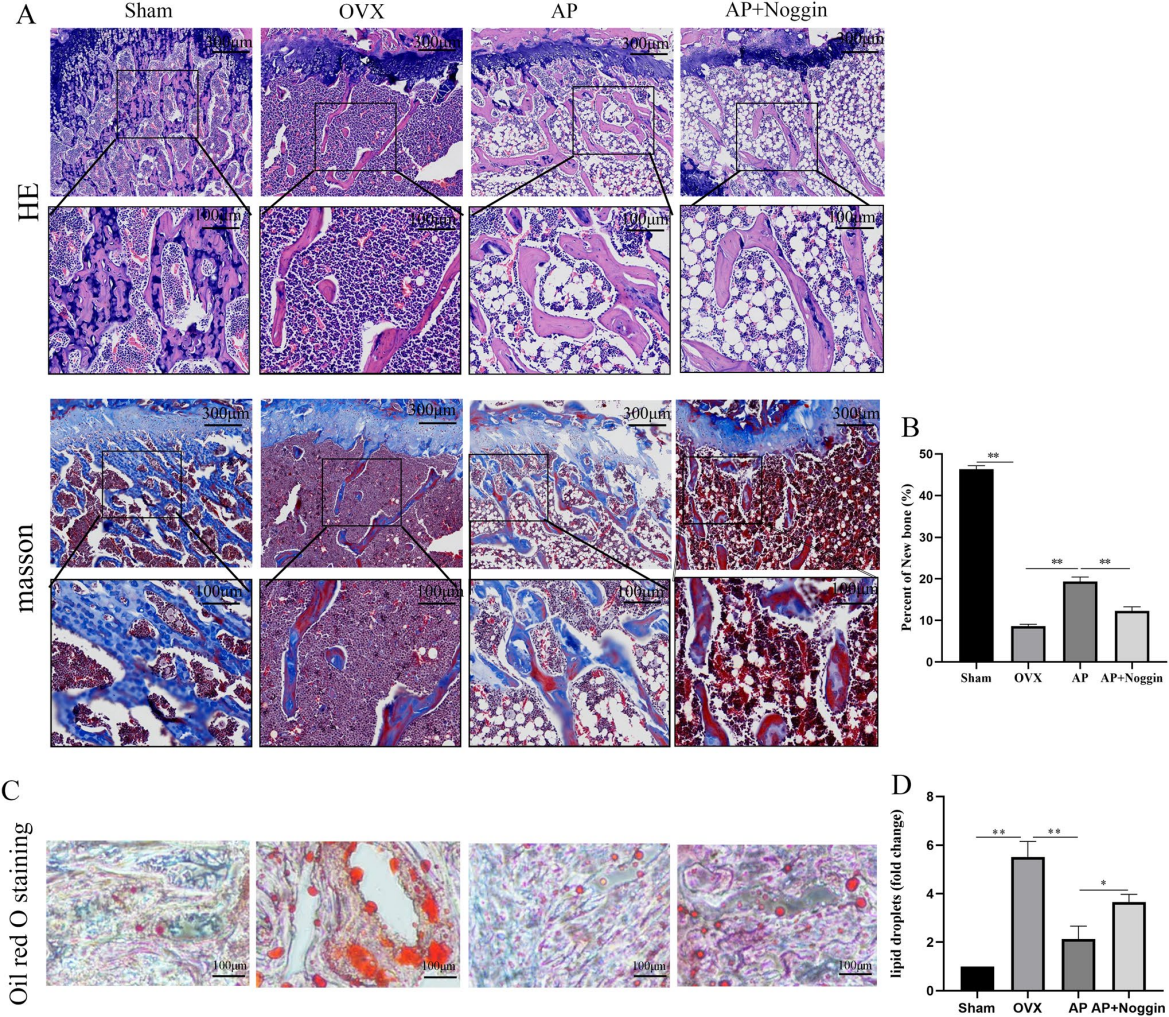
Histological and structural characteristics of the distal femur. **a** HE staining and Masson trichrome staining: Sham group, OVX group, AP group, and AP + Noggin group. **b** Statistical graph displays the rate of new bone formation in different groups. **c** Results of oil red O staining: Sham group, OVX group, AP group, and AP + Noggin group. **d** Statistical graph shows the rate of lipid droplet formation in different groups. Scale bar = 100 μm. Data are presented as the mean ± SD (n = 3). *P < 0.05 and **P < 0.01

HE staining revealed that the number of trabecular bones was lower, the trabecular space was larger, and more empty lacunas occurred in the OVX group rats compared to the Sham group, which was consistent with the micro-CT data. AP treatment improved bone mass growth in the distal femur. Masson staining revealed fresh bone production, with new bone collagen fibers in blue and mature bone in red. In comparison to the Sham group, the new bone in the OVX group was lower, and the new bone increased as the AP group. However, when Noggin was added, the protective effects of AP could be neutralized (Fig. 5a). The oil red O stain showed that the bone tissue was tightly arranged with fewer fat particles in the Sham group. More lipid droplets were seen in the bone marrow cavity in the OVX group, but the formation of lipid droplets in the marrow cavity could be significantly inhibited by AP treatment. However, this inhibition could be reversed by Noggin, a BMP2 inhibitor (Fig. 5c).

## Discussion

It has been suggested that in the context of bone loss (such as age-related osteoporosis), there is a close relationship between increased adipocytes and decreased bone formation [23]. In addition, increasing the number of osteoblasts and reducing adipocytes by targeting regulators that alter the fate of mesenchymal cells has the potential to provide a promising treatment option for osteoporosis.

This study reveals the role of AP in promoting osteogenesis and inhibiting adipogenesis in vitro and in vivo, and provides a rationale for AP in the treatment of osteoporosis. AP takes a way to regulate the differentiation of osteogenic and adipogenic lineages to prevent bone loss, as indicated by the upregulation of osteogenic protein expression and osteoblast phenotypic features, and ultimately enhances osteogenesis. To our knowledge, the effect of AP on the proliferation of ADSCs has not been clear. Therefore, we first used CCK-8 to explore the optimal concentration range of AP. The osteogenic ability of AP was then tested by ALP and ARS staining. We discovered that AP significantly improved the differentiation of ADSCs into osteoblasts. In addition, the Western blot results also indicated that AP could promote the expression of osteogenesis-related proteins COL1A1, OPN, and ALP. As a component of COL I, COL1A1 is the amplest protein in the skeleton matrix and directly participates in the mineralization and maturation of osteoblasts. ALP is a protein that is produced during osteoblast differentiation and is linked to Pi homeostasis. The Pi can be used by matrix vesicles to start crystal nucleation of extracellular matrix calcium deposits, culminating in hydroxyapatite production [23]. Osteopontin (OPN) can stimulate the adhesion, proliferation, and calcification of osteoblasts and can mediate changes in bone metabolism induced by mechanical stress [24]. We also demonstrated that AP can reduce adipocyte production during adipogenic differentiation of ADSCs. PPARγ, is the most important regulator of adipogenesis, and was inhibited in protein expression by AP.

The bone morphogenetic protein 2 (BMP2)/Smads signaling pathway has an important role to regulate osteogenesis and promote new bone formation [25]. The activation of the BMP2/Smads signaling pathway contributes to osteogenesis [26]. First, BMP2 activates its cell membrane receptor and transmits the signal to the downstream signaling molecule to induce phosphorylation of Smad1/5/8. The hetero complexes are formed by p-Smad1/5/8 and Smad4, which are translocated into the nucleus to upregulate target genes like Runx2 [27], which is an important osteogenic differentiation transcription factor that promotes the expression of osteogenic genes, such as collagen type I (col1), alkaline phosphatase (ALP), and osteocalcin (OCN) [27]. BMP signal alteration has been considered the main underlying cause of many human bone diseases [28]. We found that the protein expressions of BMP2, p-Smad1/5/8, and RUNX2 were increased in ADSCs in the OIM group and the OIM + AP group. Noggin, as an antagonist of BMP2, is particularly important in regulating BMP-mediated differentiation of mesenchymal precursor cell differentiation [29]. Noggin can directly bind to various BMPs, such as BMP-2, -4, -7, -13, and -14, thereby preventing BMPs from binding to their cell surface receptors and inhibiting the initiation of BMP signaling in target cells [30]. Thus, addition of Noggin diminishes BMP-induced osteoblastic differentiation in mesenchymal cells. We employed an inhibitor that can disrupt the BMP-2/Smads pathway to further identify the role of BMP-2/Smads in the differentiation of ADSC into osteoblasts. The main components of the osteogenic induction medium are dexamethasone, β-glycerophosphate, and ascorbic acid. Dexamethasone potently enhances the osteogenic capability of BMP-2, induces selective proliferation of BMSCs, stimulates the expression of RUNX2, ALP, OPN, and OCN, and increases the mRNA expression level of ALP [31]. Noggin is inhibitory to the BMP2 induced by osteogenesis induction medium, which has been demonstrated in other articles [32, 33]. As a result, Noggin not only inhibits the upregulated BMP2 induced by aloe polysaccharide treatment but also the BMP2 induced by osteogenesis induction medium.

The dynamic balance between bone formation and bone resorption maintains bone integrity; nevertheless, after menopause, the incidence of osteoporosis and bone loss in women increases dramatically [34]. Postmenopausal osteoporosis is the most common cause of age-related bone loss [35]. Because of its resemblance to bone loss in postmenopausal people, the OVX-induced rat model is commonly employed in osteoporosis research [36]. We are the first to assess the protective role of AP on osteoporosis in OVX-induced rats. The number of trabeculae bones in OVX rats treated with AP increased significantly, were arranged more closely, and had a more complete shape and structure compared to the OVX group, indicating that the loss of trabeculae bones induced by estrogen deficiency was significantly inhibited. At the same time, HE and Masson trichrome staining were performed on sections of the distal femur after decalcification, confirming similar results. To improve the bioavailability of Noggin, we used intraperitoneal injection administration in vivo. We believe that gavage-fed aloe polysaccharide and intraperitoneal injection of Noggin could promote osteogenesis in vivo was related to the dose of Noggin in the experiment, which makes Noggin did not completely inhibit the osteogenesis of aloe polysaccharide. However, the osteogenesis was significantly reduced and statistically significant in the AP + Noggin group compared to the AP group. Some studies have indicated that oxidative stress is high in patients with osteoporosis [37]. The imbalance between bone and fat in osteoporosis has been reported to be associated with an increasingly pro-inflammatory tissue environment with mounting oxidative stress [38]. AP has been proved to have properties, such as antioxidant, anti-inflammatory, immunomodulation in vivo. Therefore, AP may regulate oxidative stress caused by postmenopausal osteoporosis in vivo and promote the conversion of stem cells to osteogenesis.

## Conclusion

In conclusion, we conducted this study to investigate the role and mechanism of AP on osteogenic differentiation of ADSCs. In animal and cell studies, we found that AP might influence ADSC differentiation and mineralization via BMP-2/Smads. Although our experiment still has some shortcomings, AP promotes osteogenesis and inhibits lipogenic differentiation in ADSCs, suggesting that this compound has an important role in the treatment and prevention of osteoporosis. AP could be a promising treatment option for postmenopausal osteoporosis.

## Data Availability

Data and material are available after required.
